# Supplementary material for: Machine learning-assisted wearable sensing systems for speech recognition and interaction
Source: Nat Commun. 2025 Mar 10;16:2363. doi: 10.1038/s41467-025-57629-5 (PMC11894117; doi:10.1038/s41467-025-57629-5)
Supplement: Supplementary file 1 — Supplementary Information [file 41467_2025_57629_MOESM1_ESM.pdf]

Supplementary Information

Machine learning-assisted wearable sensing systems for speech recognition and interaction

Tao Liu[1], Mingyang Zhang[1], Zhihao Li[1], Hanjie Dou[1], Wangyang Zhang[1], Jiaqian Yang[1], Pengfan Wu[1], Dongxiao Li[1,*], Xiaojing Mu[1,*]

[1] Key Laboratory of Optoelectronic Technology & Systems of Ministry of Education, International R & D Center of Micro-nano Systems and New Materials Technology, Chongqing University, Chongqing 400044, China

[*]E-mail: (D. L.) lidongxiao@cqu.edu.cn, (X. M.) mxjacj@cqu.edu.cn

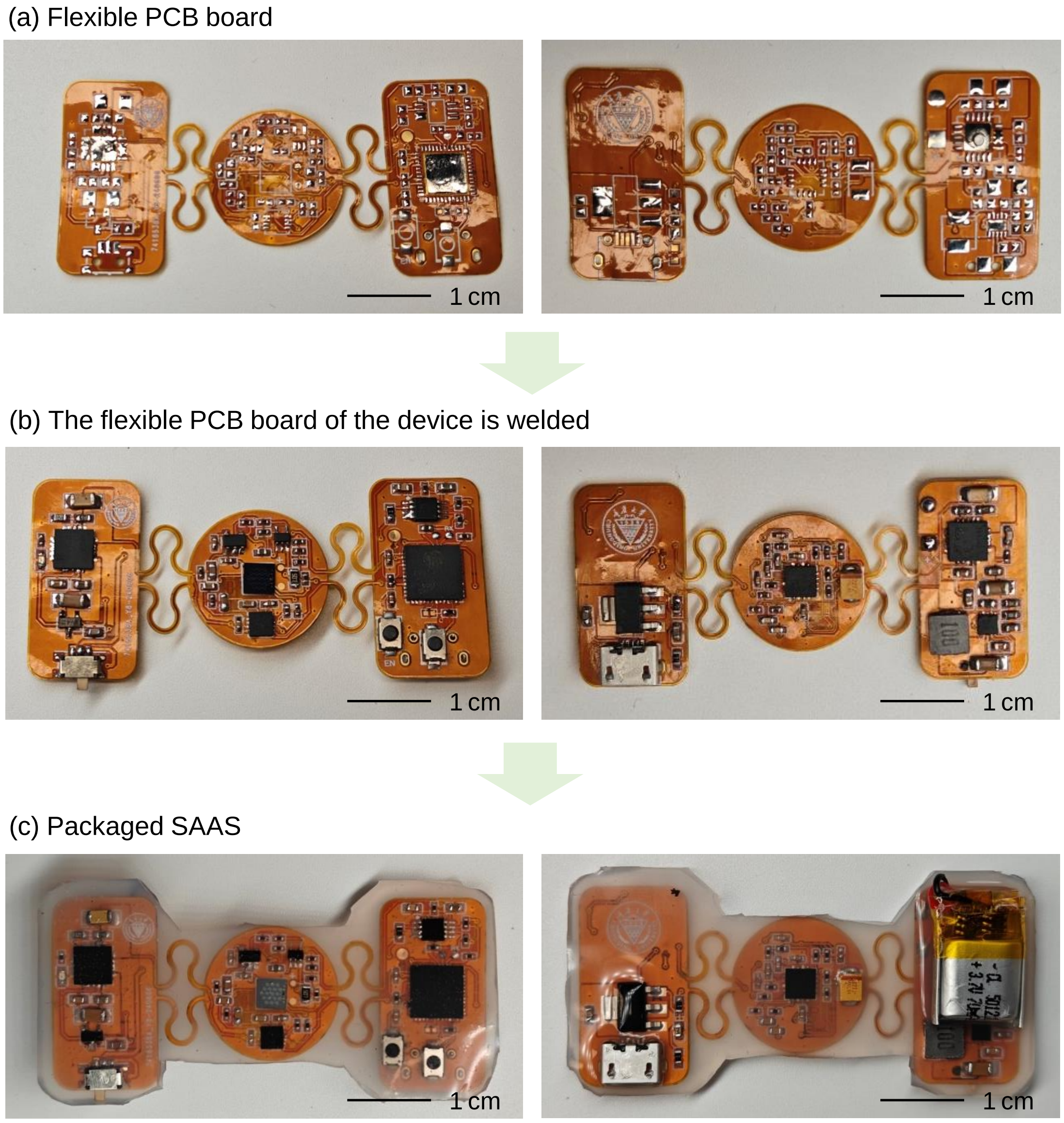

Supplementary Fig. S1 System integration and manufacturing steps. a Starting with the exposed flexible printed circuit board (FPCB). **b** Weld the chip assembly to the top. **c** After reflux, the integrated PCB is transferred to the Ecoflex-0030.

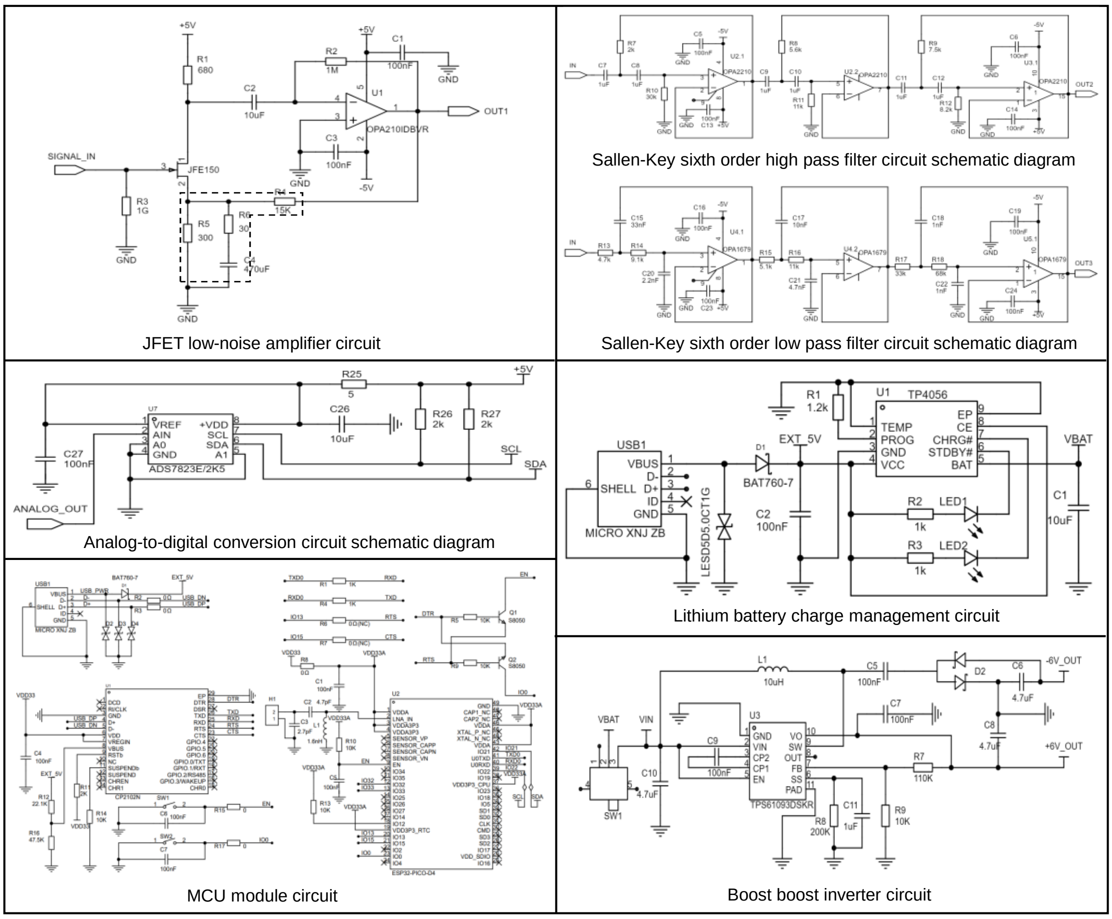

Supplementary Fig. S2 Circuit diagram of SAAS sensor.

The formula for calculating the signal-to-noise ratio is as follows,

$$SNR = 10\lg\left[\frac{\overline{P_S}}{\overline{P_N}}\right] = 10\lg\left(\frac{\overline{A_s}}{\overline{A_N}}\right)^2 \tag{1}$$

where $\overline{P_S}$ and $\overline{P_N}$ are the average power of the output signal and the average power of the sensor background noise, $\overline{A_S}$ is the average value of the voltage amplitude of the MEMS sensor detection signal, and $\overline{A_N}$ is the average value of the noise signal voltage amplitude. Through the voice test of participants, the output noise and voice signal of the SAAS are shown in the figure below, and the SNR is finally calculated to be 32.64 dB.

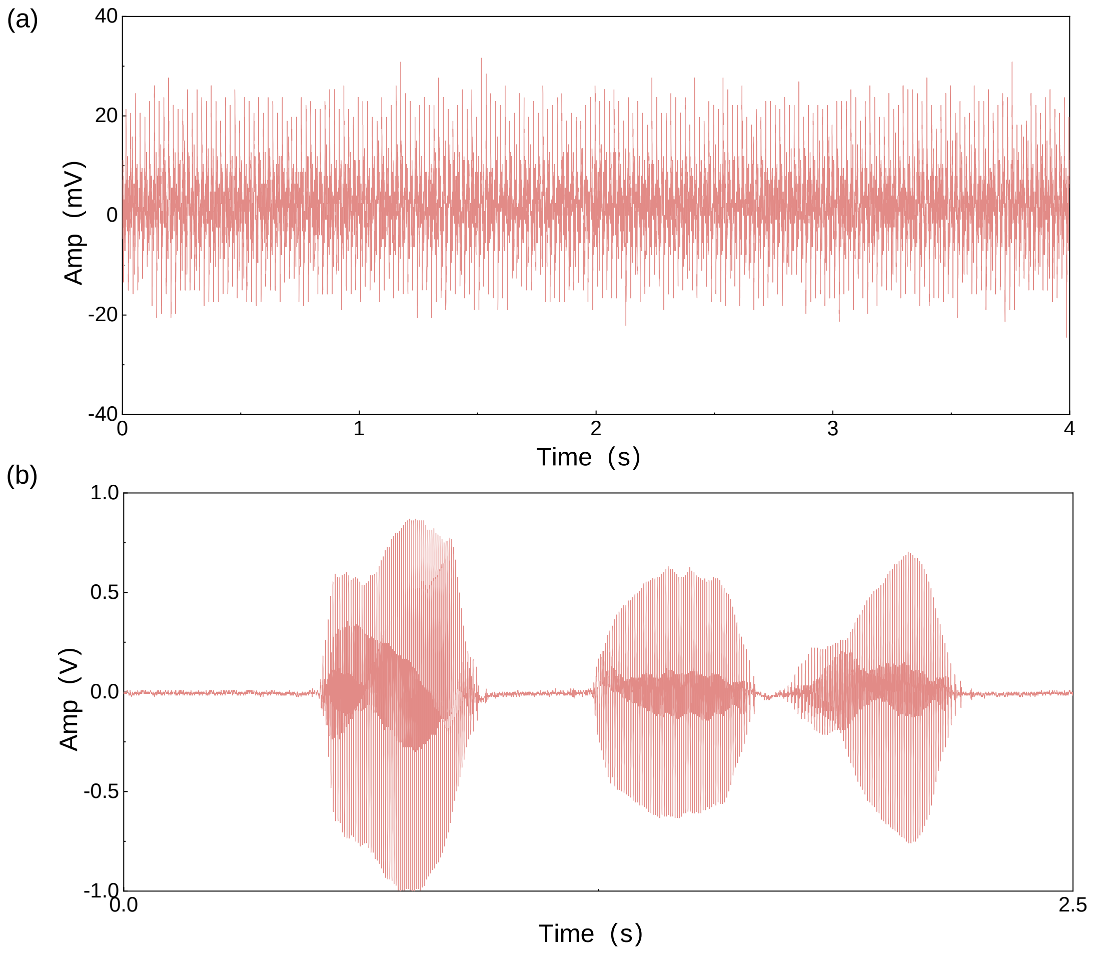

Supplementary Fig. S3 The signal-to-noise ratio test of the SAAS. a The average output noise signal voltage of the SAAS is 0.038V. **b** The average voltage of the sound signal obtained by the participants through the SAAS test is 1.5V.

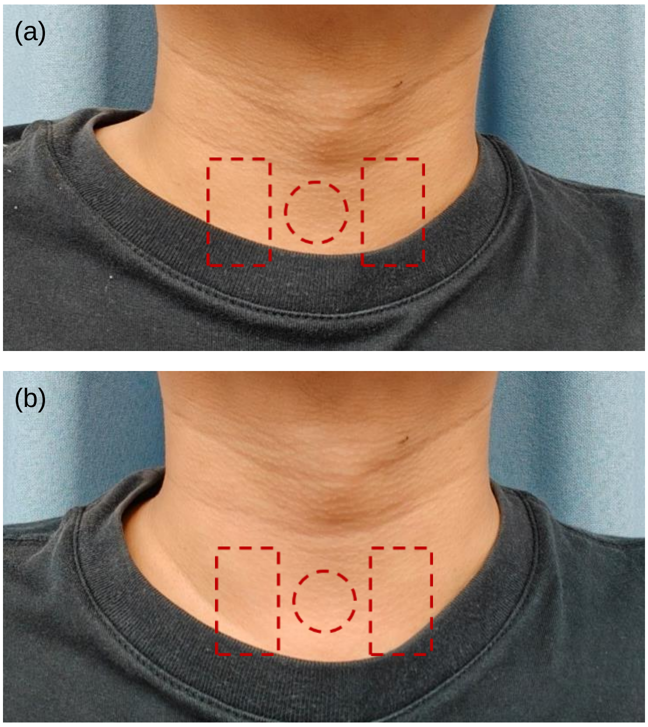

Supplementary Fig. S4. Biocompatibility and long-term wear for 5 hours. a Throat skin photo before SAAS device placement. **b** Skin photo of throat area after 5 hours using SAAS. It showed some red skin in the area where the device was attached, but no irritation occurred.

To ensure the effective electromechanical conversion of the ultrasonic transducer, the neutral axis needs to be located in the silicon device layer. The properties of each layer of materials that make up the membrane structure have a great influence on the position of the neutral axis. The position of the neutral axis is expressed as follows :

$$H_{np} = \frac{1}{2}\left[\sum \left(\frac{Y_n \left(h_n^2 - h_{n-1}^2 \right)}{1-\upsilon_n^2} \right) \Big/ \sum \left(\frac{Y_n t_n}{1-\upsilon_n^2} \right) \right] \tag{2}$$

where H_{np} is the location of the neutral axis, Y_n is the Young's modulus, υ_n is the Poisson's ratio, t_n is the thickness of the n layer, and h_n is the z-axis position of the upper part of the *n* layer.

$$f_r = \frac{(3.2)^2}{2\pi a^2}\sqrt{\frac{D}{\mu}} \tag{3}$$

$$D = \frac{1}{3}\sum Y_n \frac{(h_n - H_{np})^3 - \left(h_{n-1} - H_{np}\right)^3}{3\left(1-\upsilon_n^2\right)} \tag{4}$$

$$\mu = \sum \rho_n t_n \tag{5}$$

where a is the radius, D is the stiffness of the plate, μ is the mass per area of the plate, and ρ_n is the density of the different materials. Considering the co-design of sensor sensitivity and flatness, the resonant frequency is finally determined to be 300 kHz.

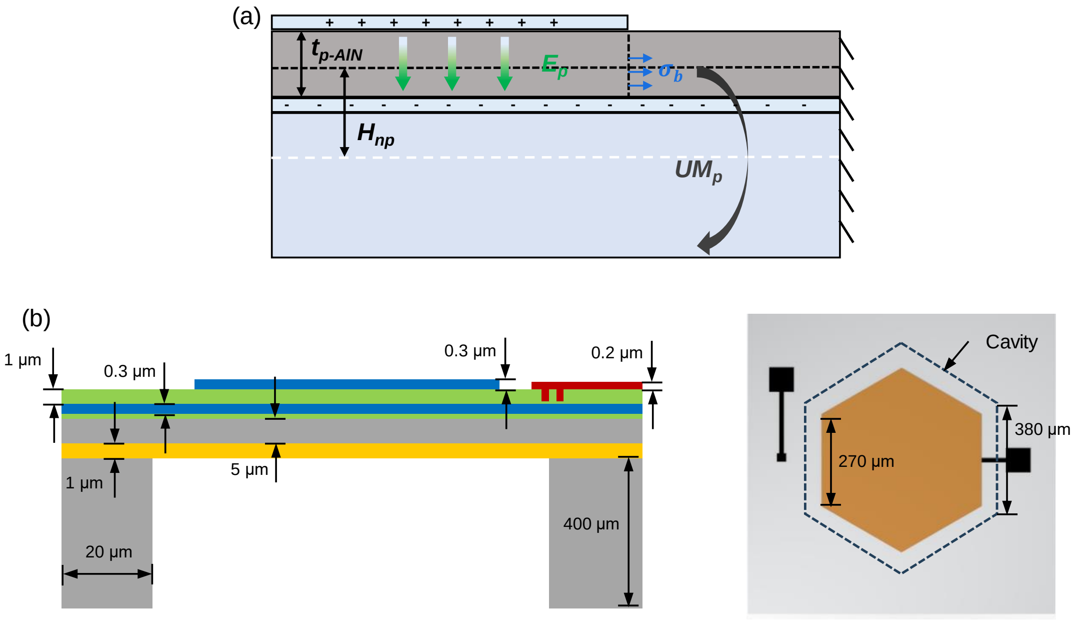

Supplementary Fig. S5 The working principle and parameter design of PMUT. a Working principle diagram of PMUT**.** Here, the cavity causes the sensor to generate a bending moment about the neutral axis in the stress gradient along the thickness of the film in the area covered by the electrode, causing the film to vibrate. **b** Structure parameters of PMUT.

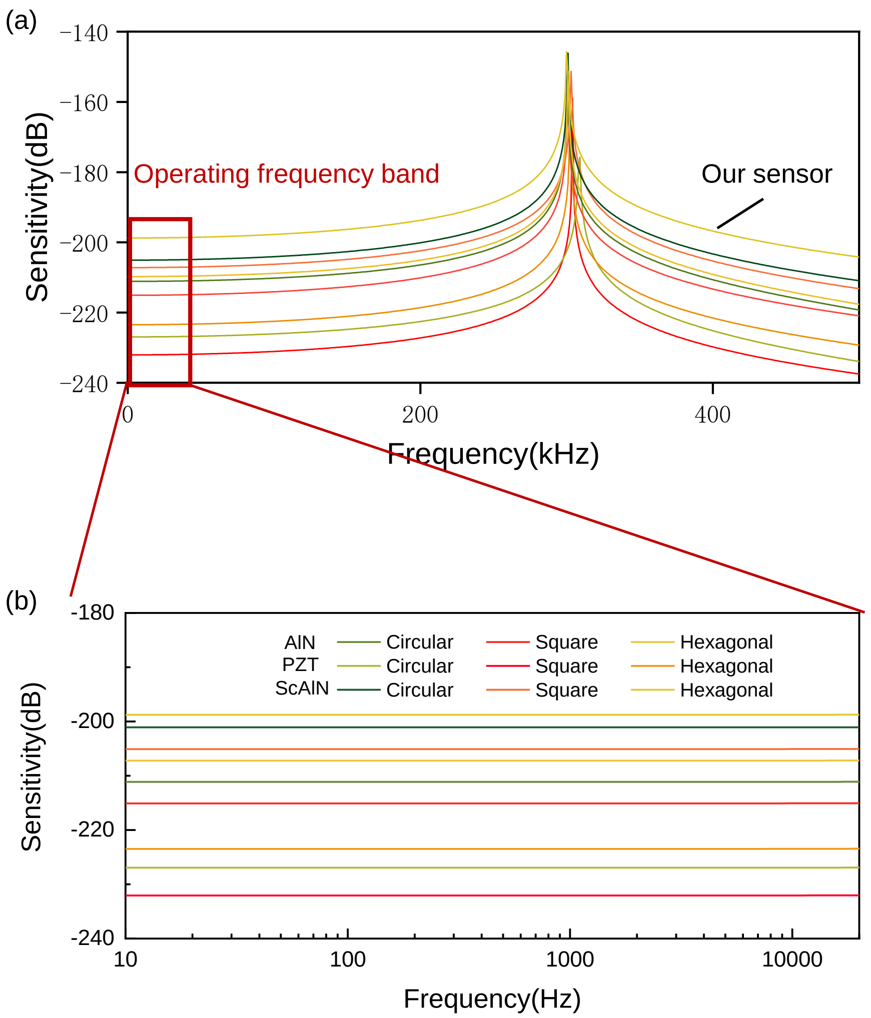

Supplementary Fig. S6 Finite element simulation of frequency response curve of MEMS acoustic sensor. a Frequency response curve of MEMS sensor in 0 – 500 kHz band. **b** Local amplification frequency response curve of MEMS sensor in 10 – 20 kHz band. The resonant frequency of our sensor in the air domain is 300 kHz, which can ensure the sensitivity of the sensor and also ensure its flatness in the wide band.

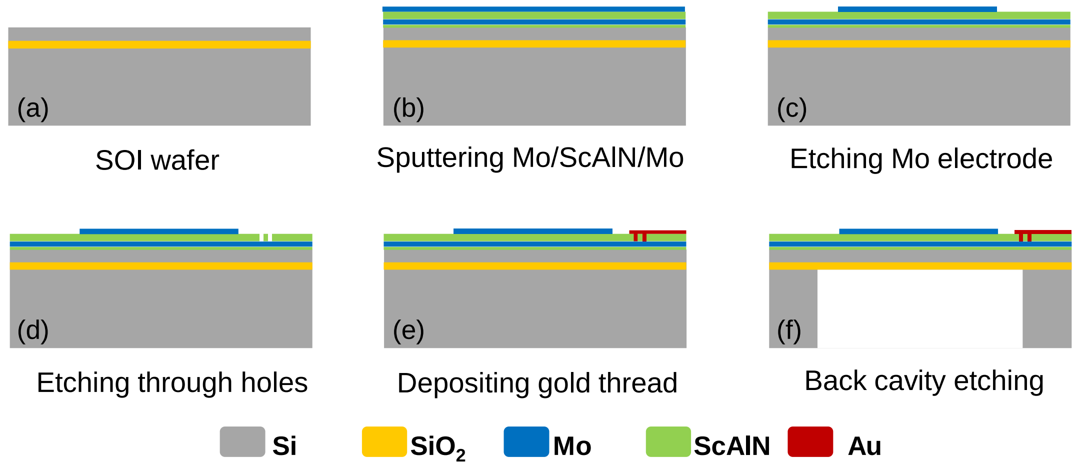

Supplementary Fig. S7 The fabrication process of the acoustic sensor. a The processing process of the MEMS sensor starts with the cleaning of the silicon on insulator (SOI) wafer (6N600-1-5N, Okmetic). The SOI wafer consists of a 5 μm silicon device layer, a 1 μm buried silicon oxide layer, and a 400 μm silicon substrate. **b** A ScAlN seed layer of about 100 nm was grown through atomic layer deposition (ALD) (NLD-4000, Nano-master) to reduce the surface roughness of subsequent film deposition, thereby improving the structure and morphology of Mo and c-axis oriented ScAlN. Physical vapor phase Deposition (PVD) (Sigma, SPTS) was then used to sequentially deposit 0.3 μm Mo, 1 μm ScAlN, and 0.3 μm Mo on the ScAlN seed layer. **c** In the subsequent patterning step, inductively coupled plasma etching (ICP) (GSEC200, NMC) was used to form the top Mo electrode pattern. **d** ScAlN was then etched to form the bottom electrode lead-out hole. **e** Next, 200 nm Au was deposited by magnetron sputtering (MS150X-L, FHR) and metal leads and pads were formed by the lift-off method. **f** Finally, deep reactive ion etching (DRIE) (Omega LPX Rapier, SPTS) was performed from the back of the SOI to release cavities and form thin film vibration structures.

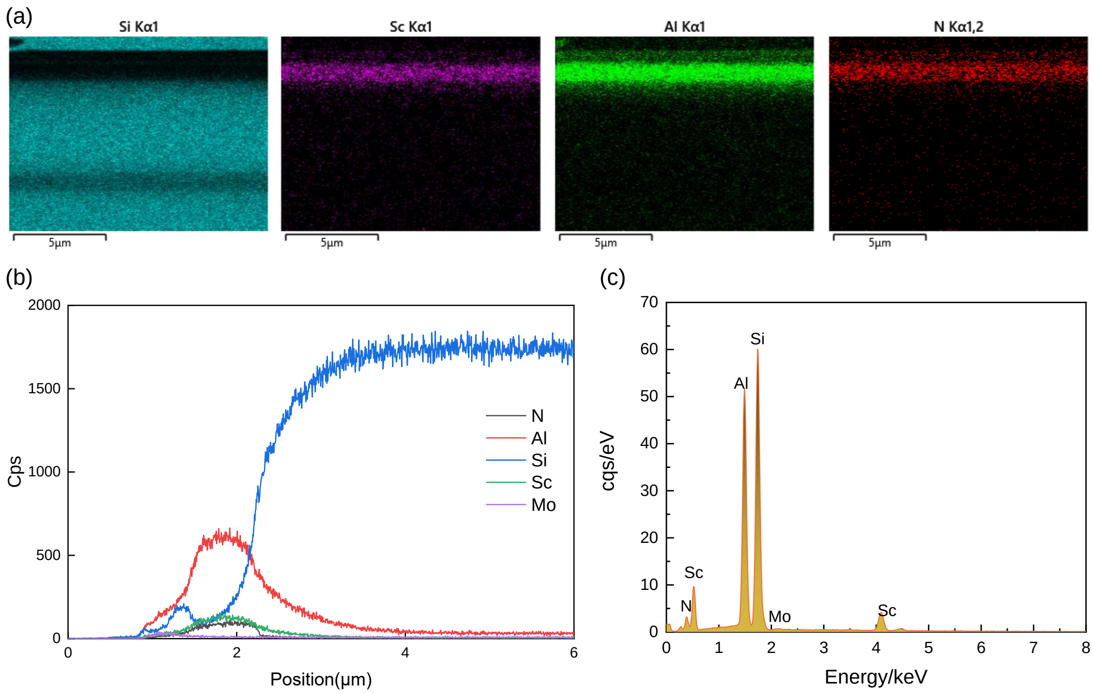

Supplementary Fig. S8 Grooves about 20 μm × 20 μm × 10 μm were opened on the surface of ScAlN thin films using a focused ion beam (FIB, Scios dual-beam scanning electron microscopy /FIB system). The scandium concentration on ScAlN cross section was measured by energy dispersive X-ray spectrometer at 15 keV beam energy. Energy-dispersive X-ray spectroscopy (EDS) scanning data is shown in the figure. The results show that the scandium concentration remains consistent throughout the film thickness. **a** Corresponding EDS mapping of four key elements for observing Si and ScAlN layers. **b** EDX results over the line scan. **c** EDS results.

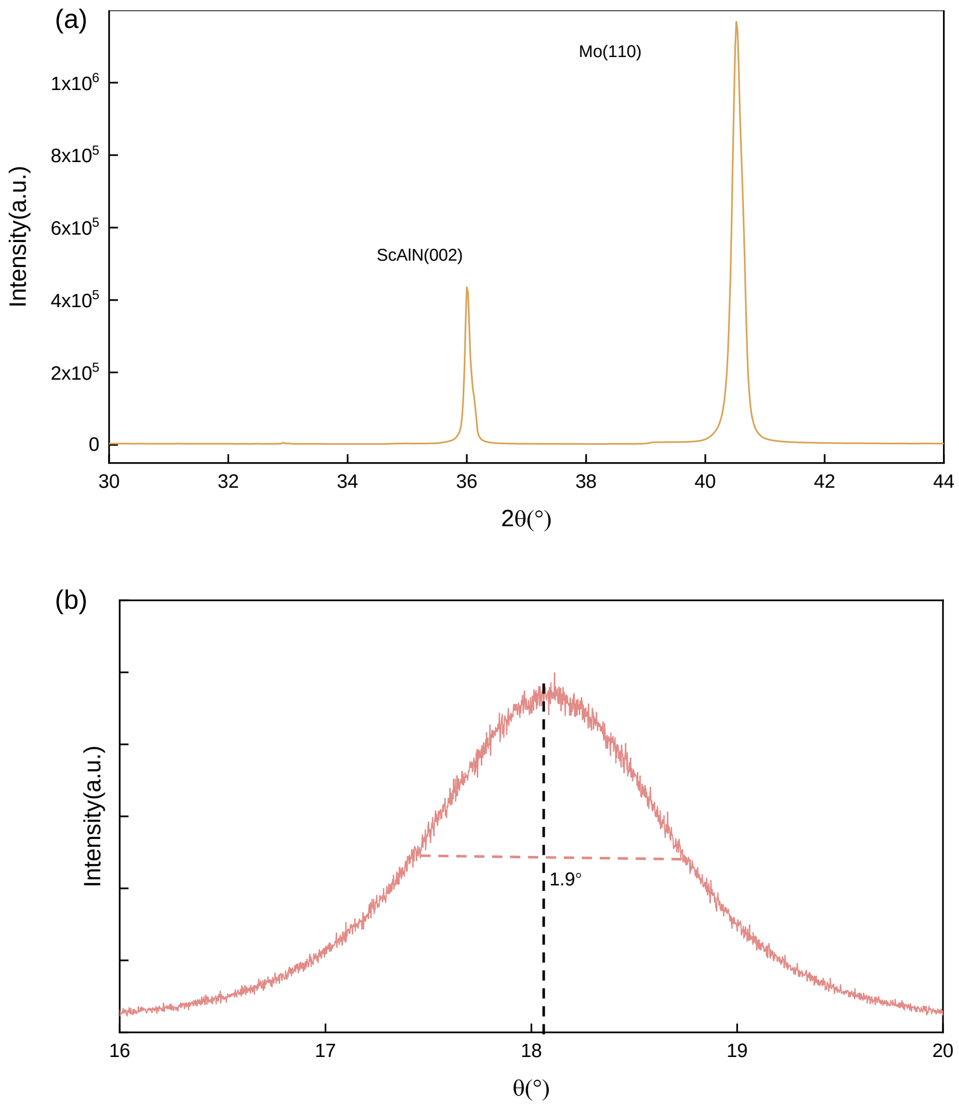

Supplementary Fig. S9 The crystal structure of ScAlN was studied by X-ray diffraction (XRD). **a** XRD characterization of ScAlN thin films. **b** Measured the rocking curve of the peak ScAlN(002). The FWHM of ScAlN film is 1.9°, indicating that the c axis of ScAlN film is well arranged, indicating good piezoelectric properties.

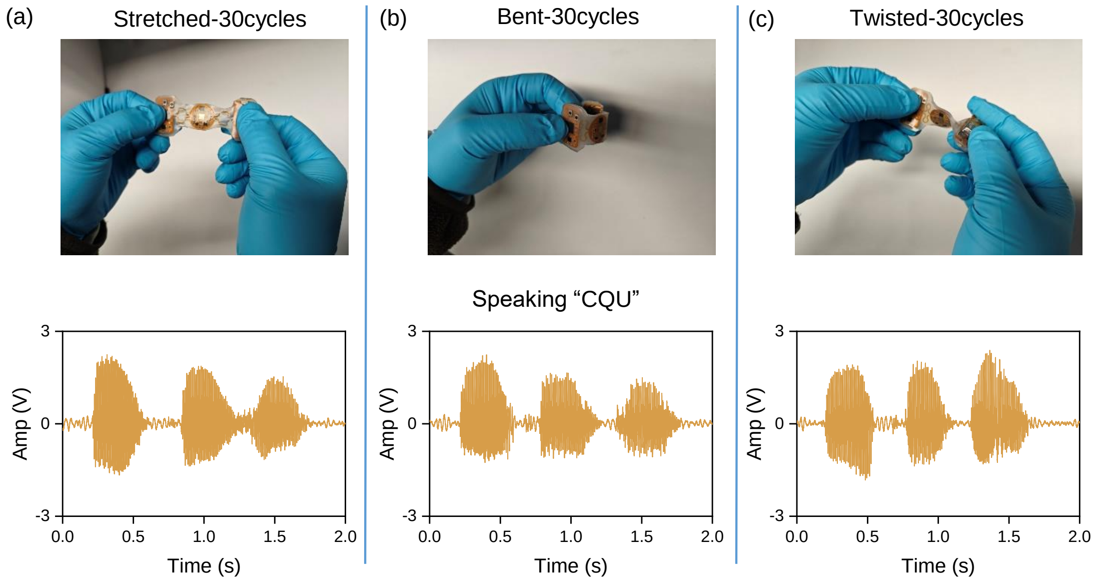

Supplementary Fig. S10. The electromechanical performance of the SAAS after testing in various mechanical deformation cycles. a The SAAS collected the signal of “CQU” from participants after 30 stretching cycles. **b** The SAAS collected the signal of “CQU” from participants after 30 bending cycles. **c** The SAAS collected the signal of “CQU” from participants after 30 twisting cycles.

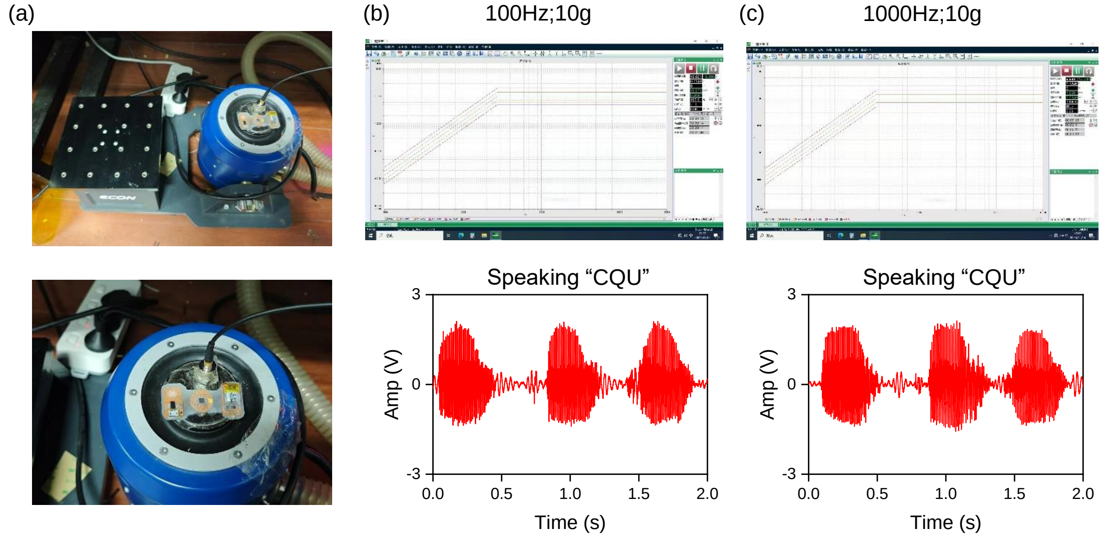

Supplementary Fig. S11. Structural stability test of PMUT. a Structural stability test platform. **b** Set the vibration table at the parameters of acceleration 10g and frequency 100 Hz, and vibrate for 30 minutes. The “CQU” voice signal is then collected by the sensor. **c** Set the vibration table at the parameters of acceleration 10g and frequency 1000 Hz, and vibrate for 30 minutes. The “CQU” voice signal is then collected by the sensor.

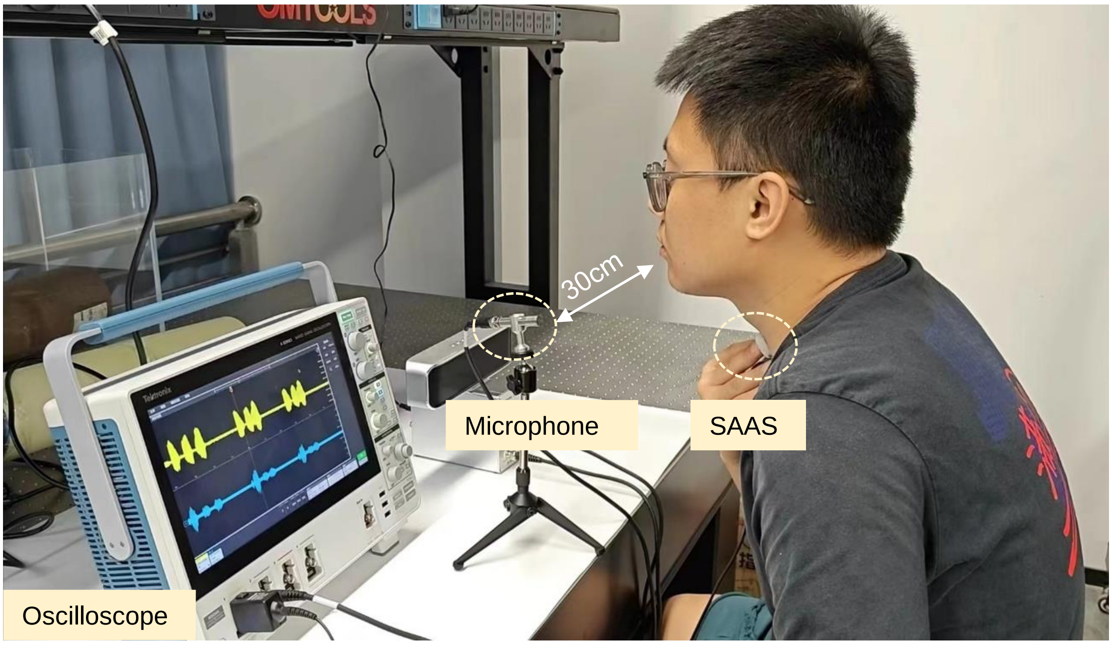

Supplementary Fig. S12 Experimental device for contrast experiment. Our sensors are attached to the skin of the human neck. The output signals from our sensors and commercial reference microphones are simultaneously transmitted to the oscilloscope. The oscilloscope displays real-time output waveforms and uses commercial microphones to measure the volume and frequency of the human voice. An amplifier is attached to a commercial microphone to get enough electrical signals from the human voice. (In order to display the signal on the oscilloscope, our sensors are wired).

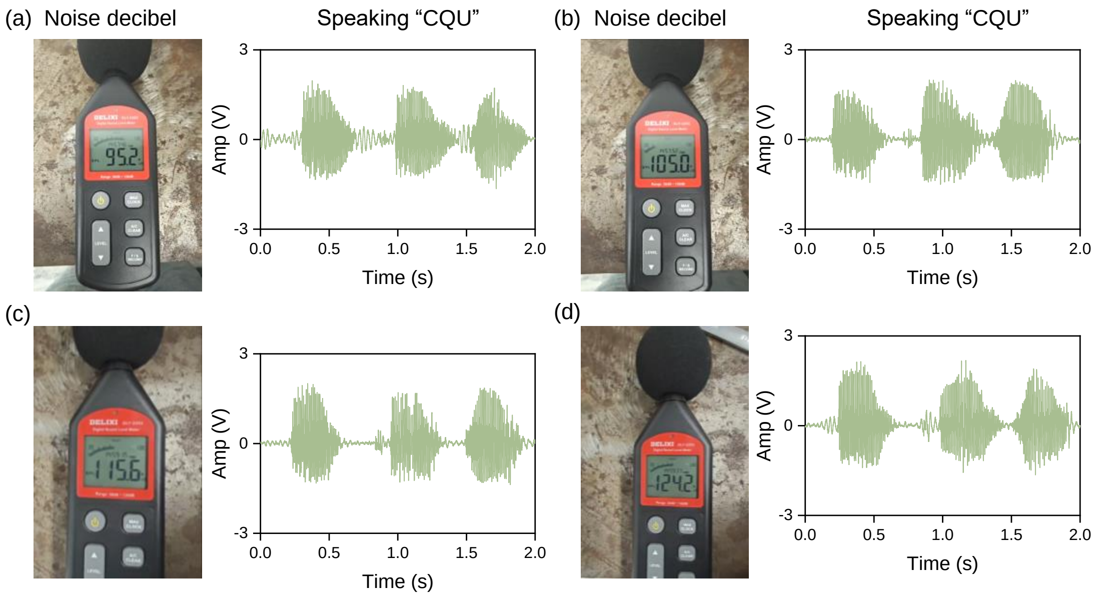

Supplementary Fig. S13. Sensor testing in different decibel noise environments. a Voice signal collected by sensor in 95 dB noise environment. **b** The voice signal collected by the sensor in the 105 dB noise environment. **c** Voice signal collected by sensor in 115 dB noise environment. **d** Voice signal collected by sensor in 125 dB noise environment.

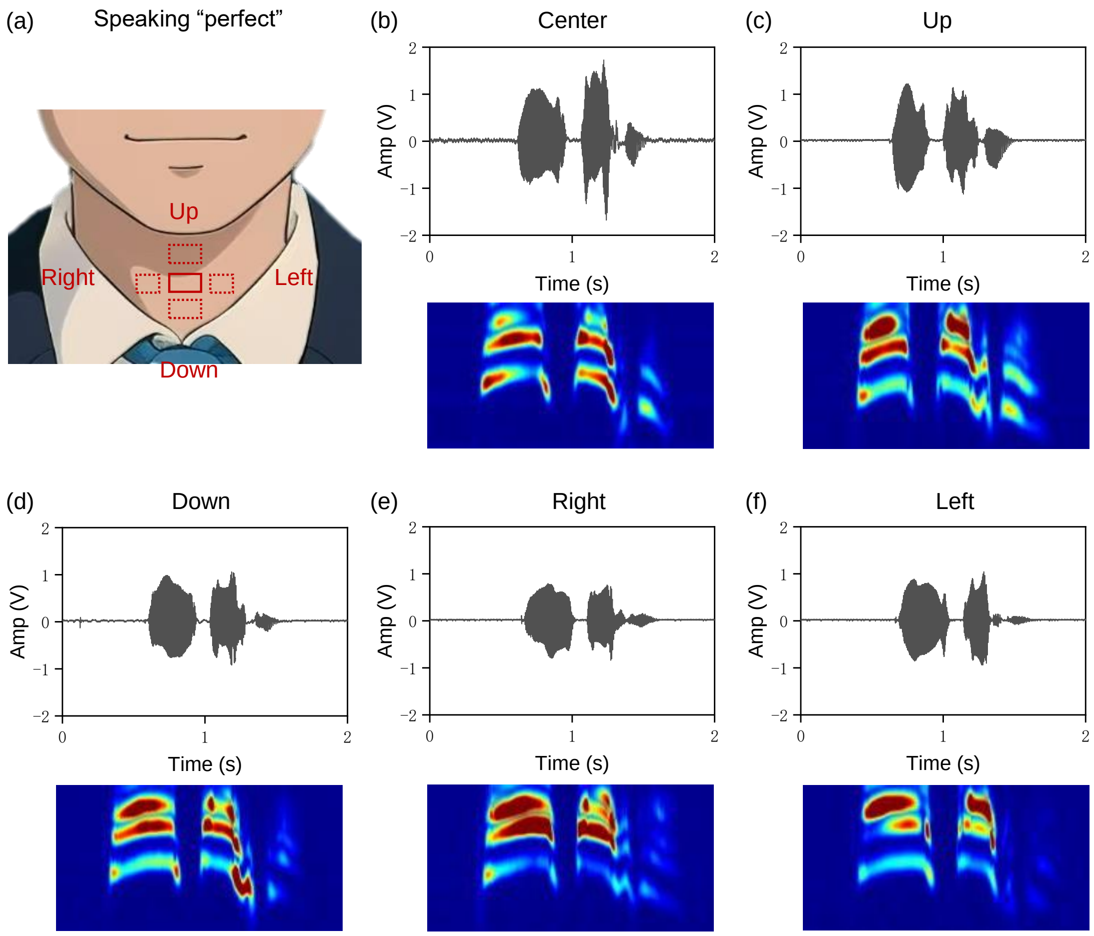

Supplementary Fig. S14 The SAAS test comparison in different wear positions. a Diagram of the location of SAAS attached near the throat. When participants say the word "perfect", SAAS captures a time-domain and time-frequency map of the signal attached to the center **b**, up **c**, down **d**, right **e** and left **f** of the throat.

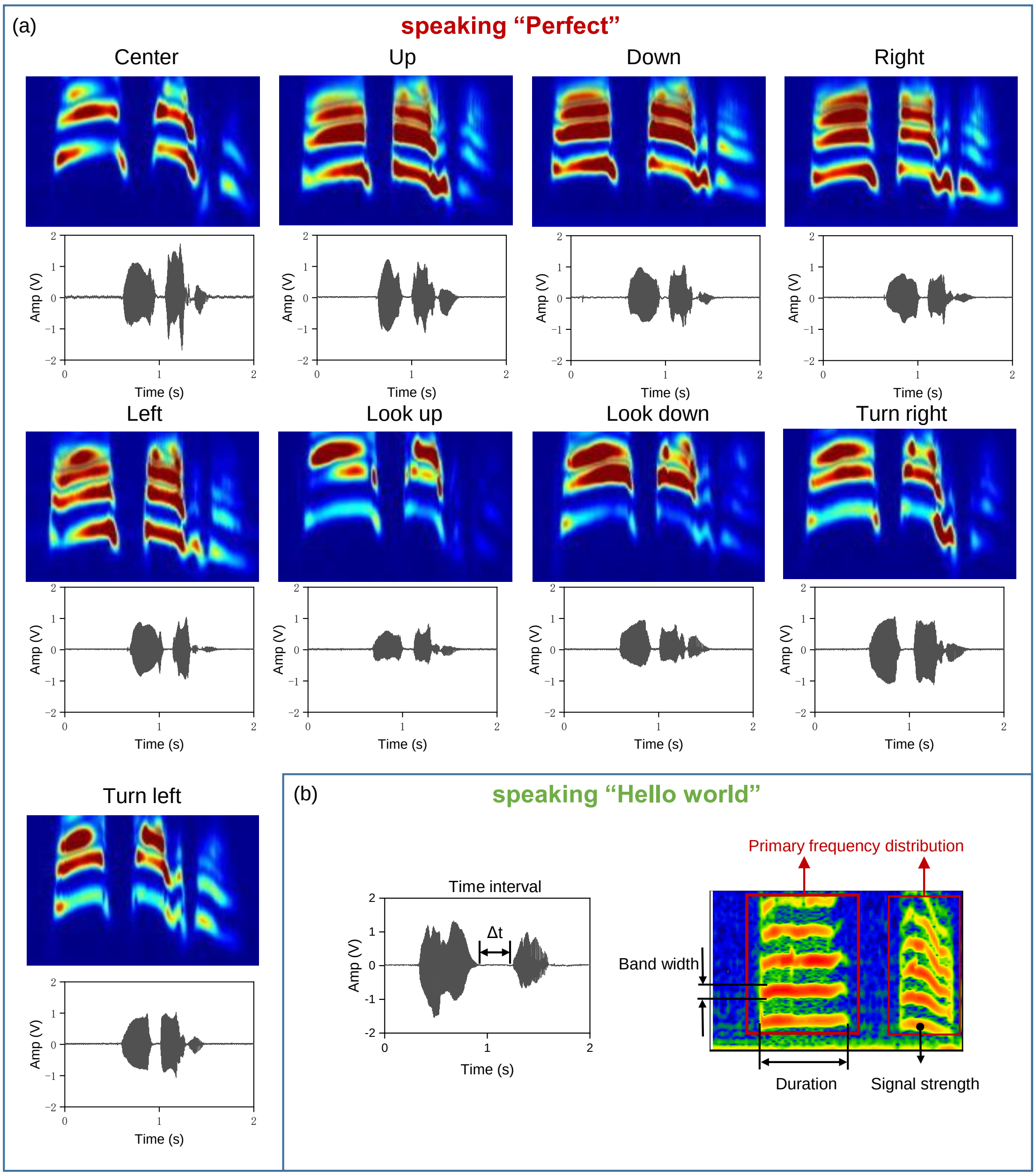

Supplementary Fig. S15 SAAS testing in different positions and actions. a Time domain and frequency domain signals for 9 usage conditions. **b** The time-spectral characteristics of the word "hello world" signal.

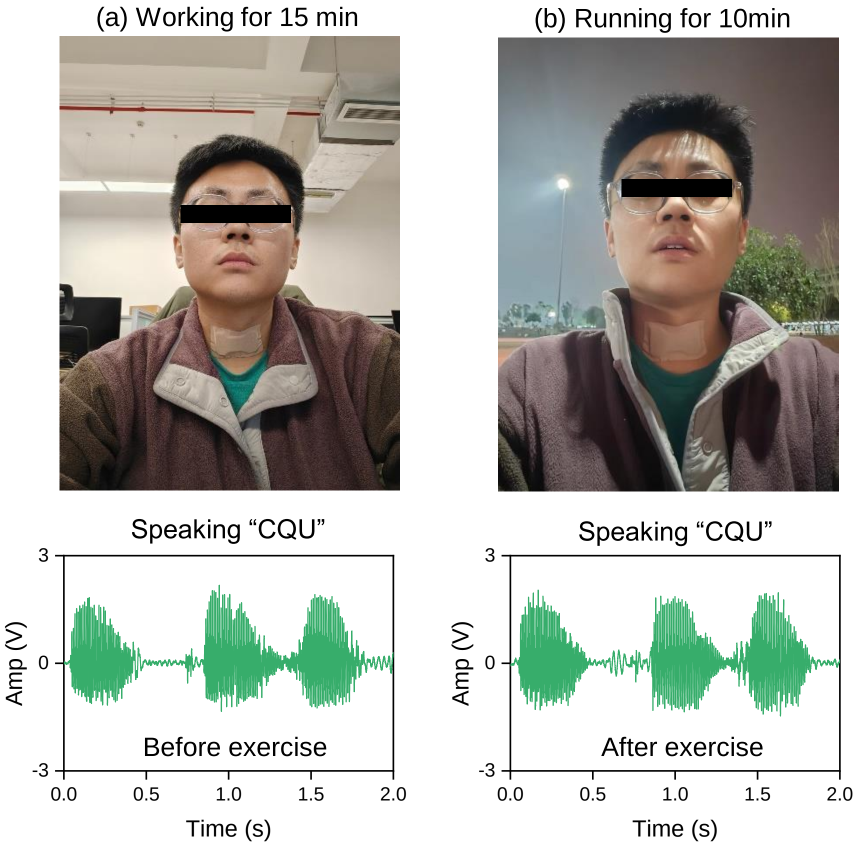

Supplementary Fig. S16 Wear stability test of the device. a Participants wearing SAAS while working in the lab for 15 minutes and the signals collected. **b** Participants wearing SAAS while running on the playground for 10 minutes and the signals collected.

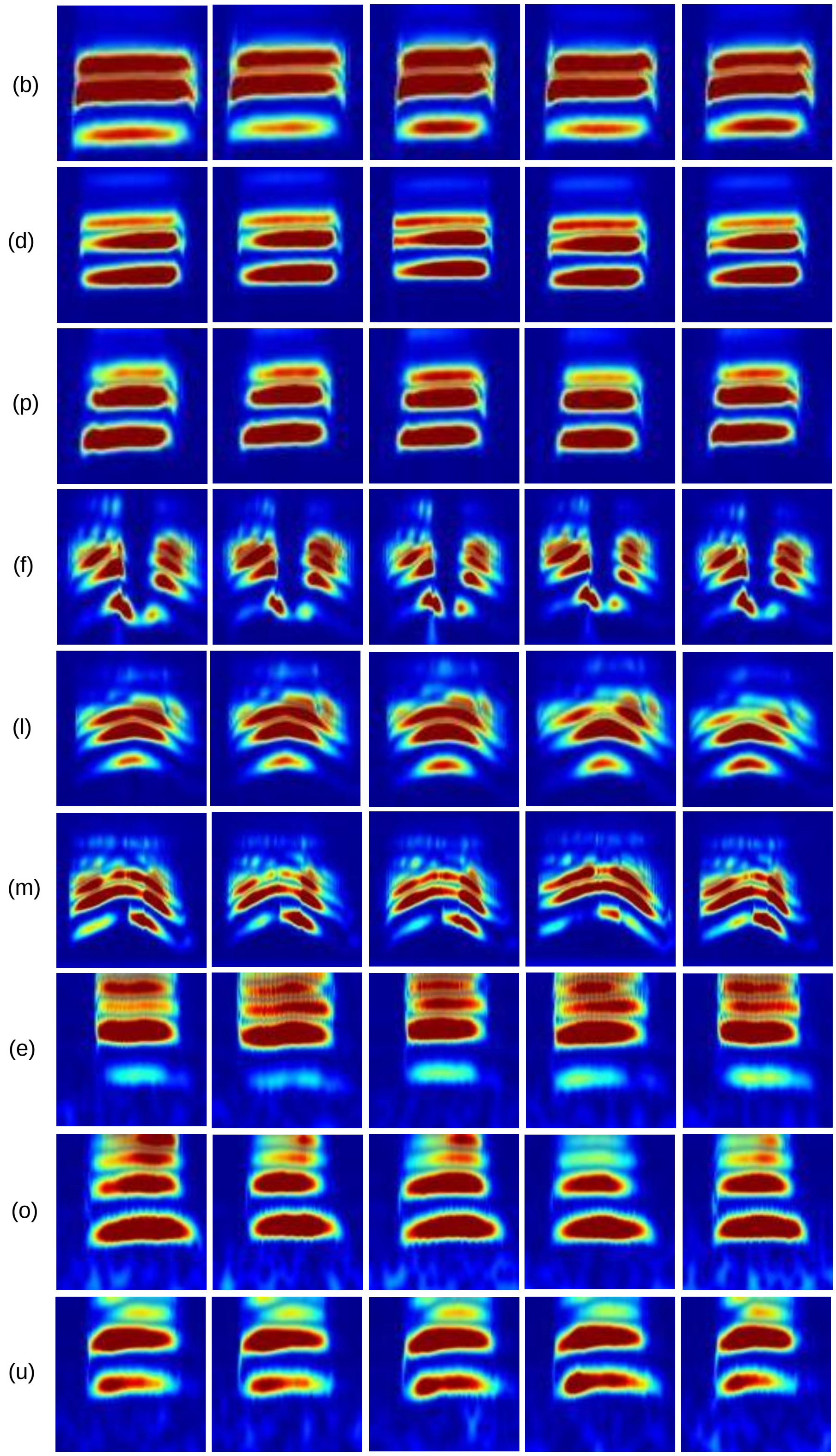

Supplementary Fig. S17. Typical local sample representation of the phoneme dataset. Redundant information (such as coordinate axis labels) was removed to facilitate feature extraction by convolutional neural networks. Different phonemes have specific discernible characteristics in low and middle-frequency bands. The pronunciations of the letters b, d, p, f, l, m, e, o, and u correspond to five time-frequency diagrams in each row. For ease of display, these time-frequency diagrams are randomly selected from a large number of test data.

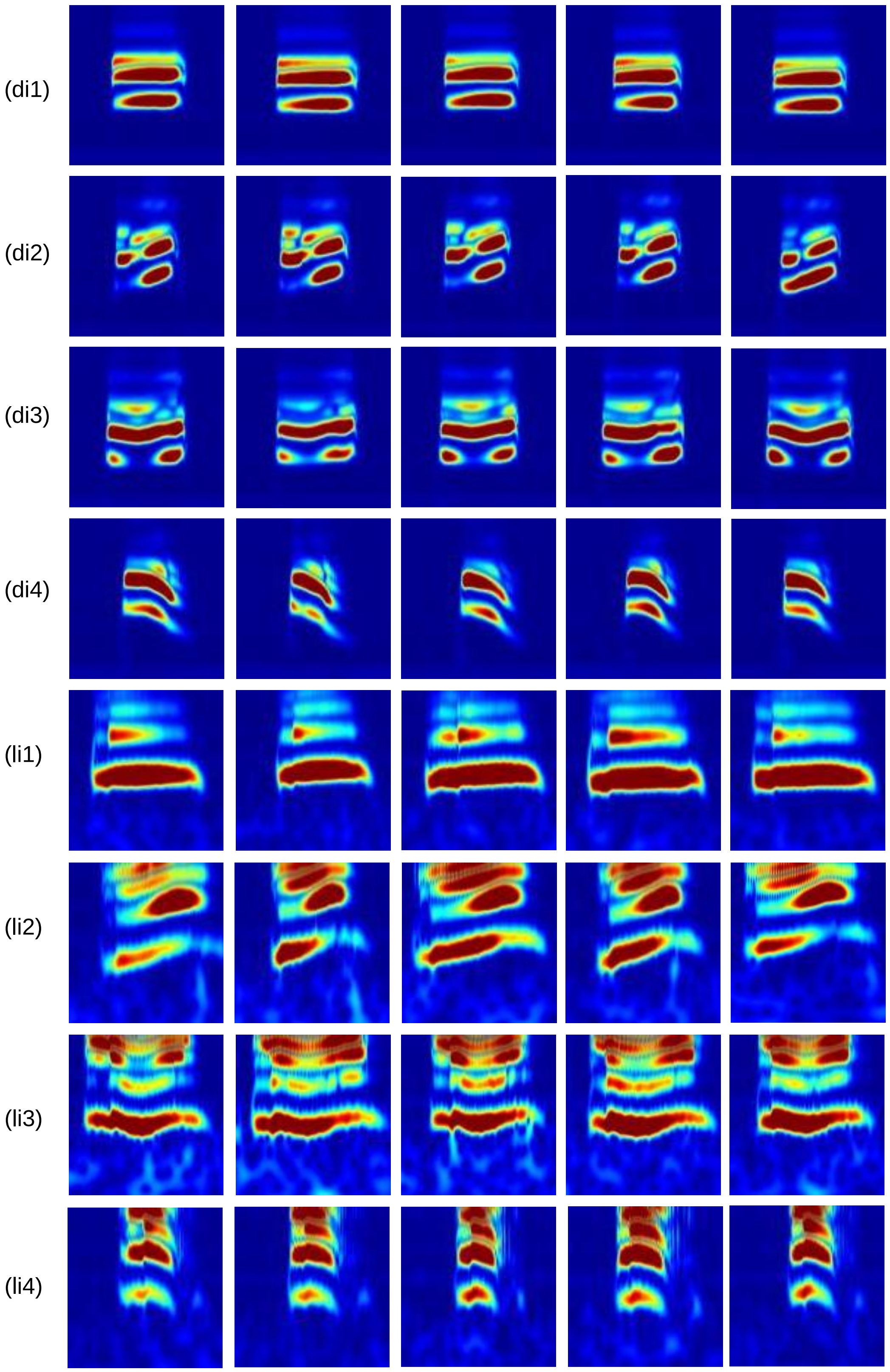

Supplementary Fig. S18. Typical local sample representation of the tone dataset. The four tones of mandarin have a different amplitude of throat movements and time distribution of intensity. Among the four tones, the fourth falling tone is the shortest and faintest.

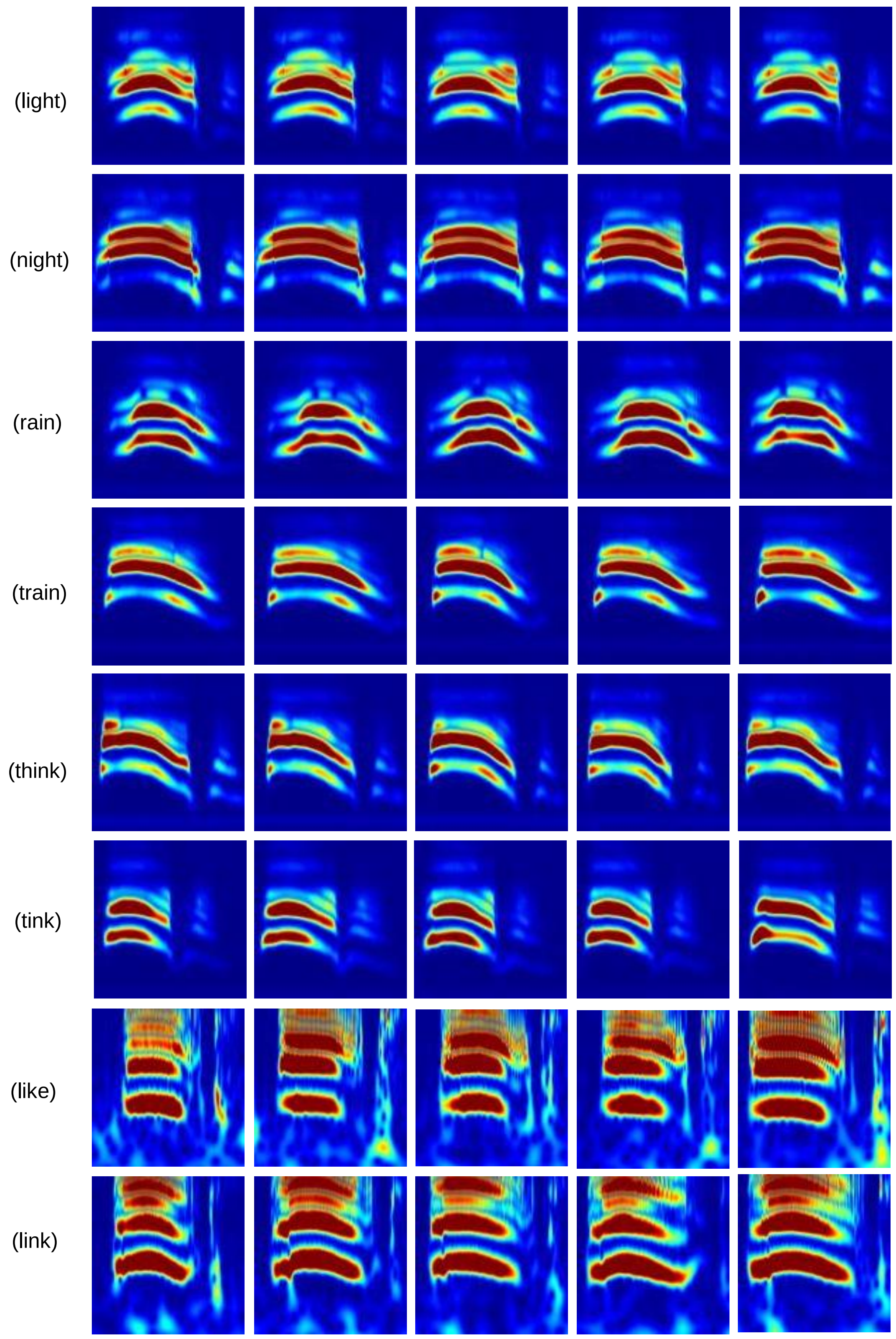

Supplementary Fig. S19. Typical local sample representation of the word with similar pronunciations dataset. The words with the same pronunciation have a certain similarity in the overall characteristics of the time-frequency graph, but the intensity distribution at different frequencies and times has its own unique characteristics.

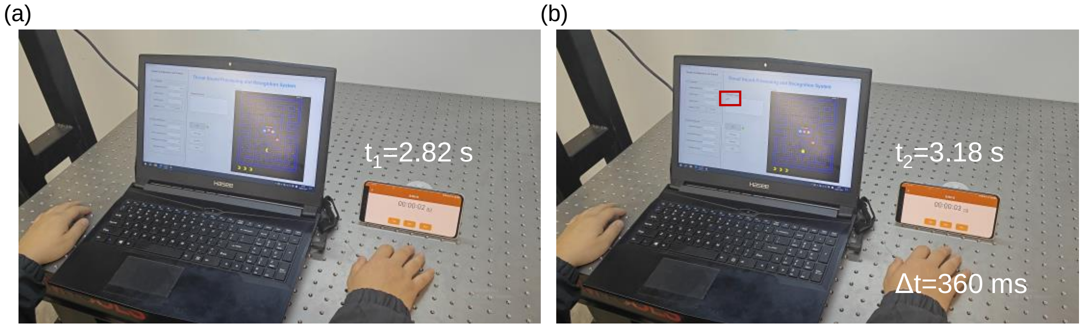

Fig. S20. Test the signal lag of the device. a The moment when the voice command ends. **b** The moment when the command is executed.

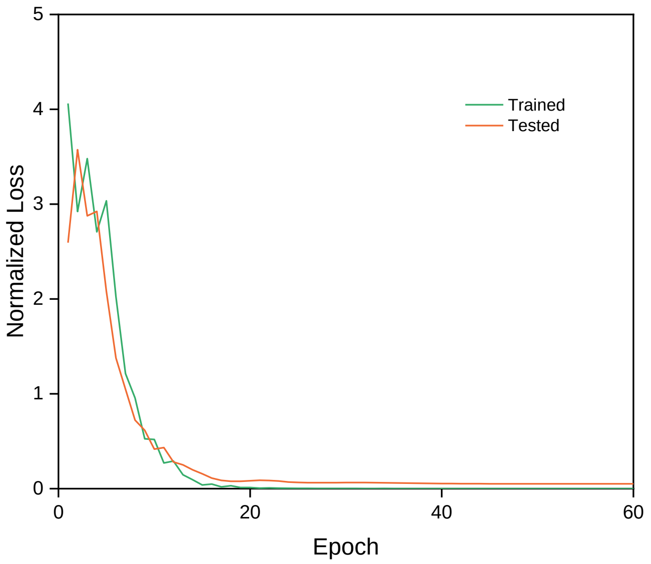

Supplementary Fig. S21 Normalized loss of training and test data during 60 epoch iterations.

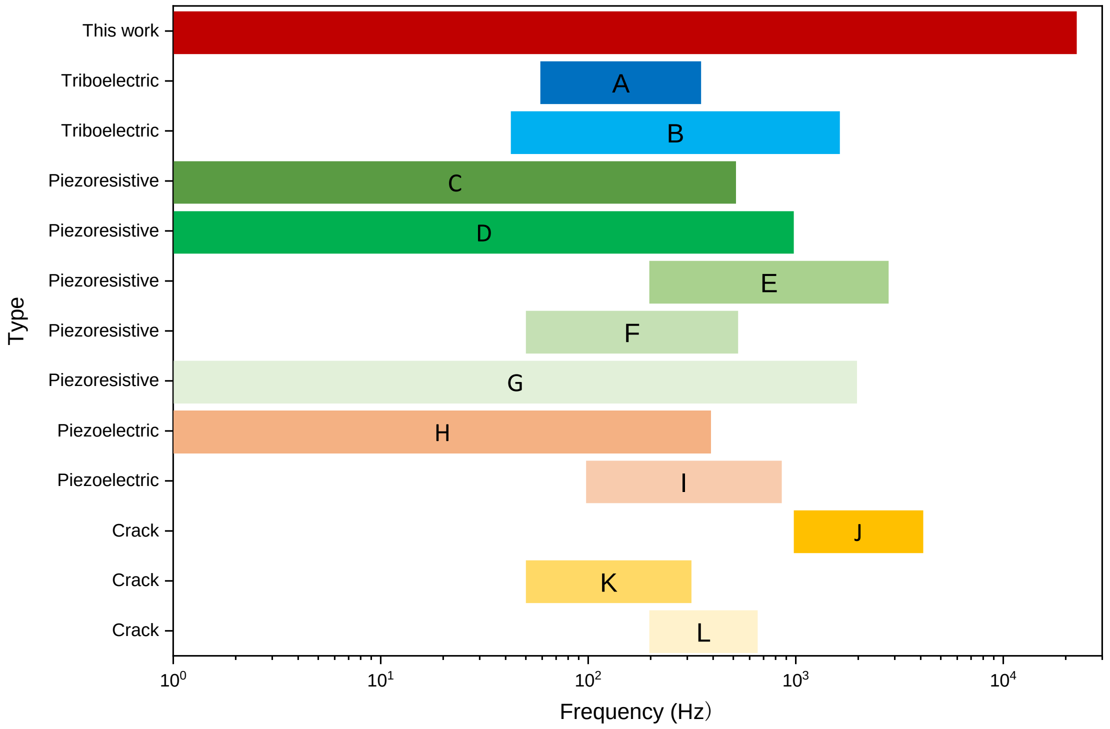

Supplementary Fig. S22 A comparison of response frequencies. A–L correspond to refs. 1–12, respectively.

(a) Stand-by current (b) Working current

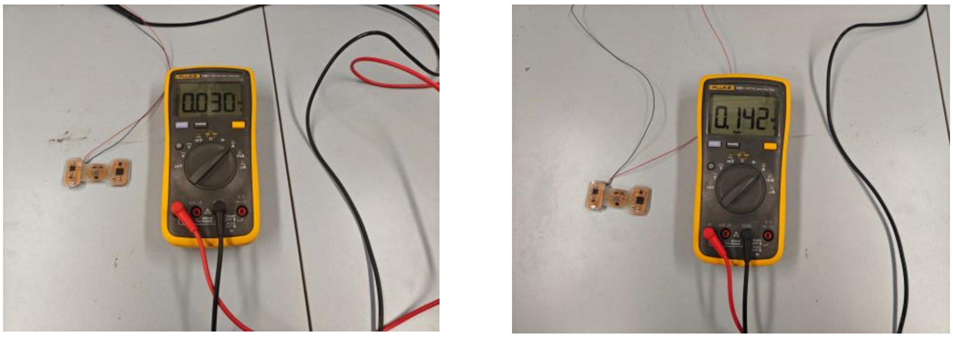

Supplementary Fig. S23. Power consumption testing of device. a Current in standby state**. b** Current of the data transmission state.

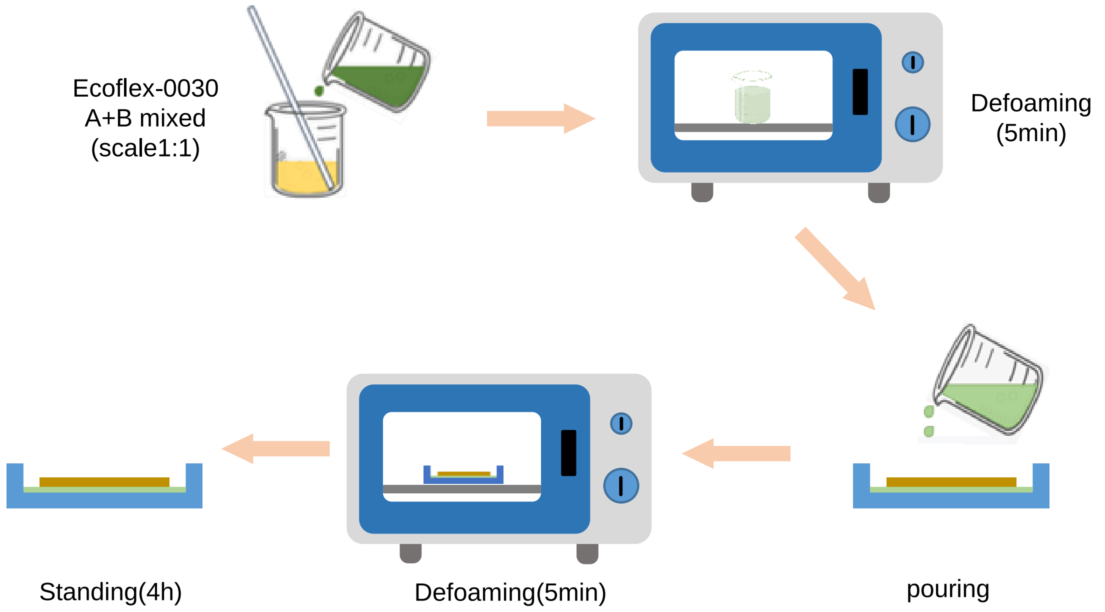

Supplementary Fig. S24 The schematic diagram of the SAAS flexible package preparation process.

Table S1 Parameter comparison of PMUT stacking materials.

	**Mo**	**ScAlN**	**Si**	**SiO_2**
Young modulus (Gpa)	312	230	170	60
Density (kg/m^3)	10200	3290	2329	2200
Poisson's ratio	0.3	0.26	0.28	0.17

Table S2 Sensitivity comparison of piezoelectric films.

**Comparison**	**PZT**	**ZnO**	**AlN**	**AIScN**
Density [kg/m^3]	~7500	~5680	-3260	~3306
Longitudinal Velocity [m/s]	~4600	~6350	~11300	~9935
Transverse velocity [m/s]	~2000	~2720	~6000	~5700
Theral Conductivlty [W/(m·K)]	-1.8	60	280	NA
TCF [ppm/°G]	NA	-120	-50	-80
e_{31} [C/m^2]	-10	-1	-1,05	-1.6
ε_{33}	1000	10.9	10..5	12
Rx Sensitivity $\propto d_{31}/\varepsilon_{33}$	-0.01	-0.092	-0.1	-0.133

Table S3 Comparison of different sensor.

Sensing mode	Flexibility	Systemic integration	Wireless	Monitoring	Trained by ML	Ref.
Resistive changes	Semi-flexible	N	N	Sound signal	N	13
Resistive changes	Full-soft	N	N	Touch and neck movement	Y	14
Strain, EMG	Full-soft	N	N	Voice signal	Y	15
Strain	Full-soft	N	N	Voice signal, Muscle motion	Y	16
Strain, EMG	Semi-flexible	N	Y	Muscle activity, throat movements	N	17
Acceleration	Semi-flexible	Y	Y	Swallowing, respirations	N	18
Acceleration	Semi-flexible	Y	Y	Artifact-canceled physiological	N	19
Acceleration	Semi-flexible	Y	Y	Physiological processes, body motions	N	20
Acceleration, EMG	Full-soft	N	N	Electrophysiological signals	Y	21
Triboelectric	Semi-flexible	N	N	Voice signal	N	22
Resistive changes	rigidity	Y	Y	Muscle motion	N	23
This work	Semi-flexible	Y	Y	Voice signal, Muscle motion	Y	/

N: no, Y: yes, EMG: Electromyogram, ML: Machine-learning

Table S4 Essential parameters used in model training.

**ResNet18**	
Momentum	0.9
InitialLearnRate	0.001
LearnRateSchedule	None
L2Regularization	0.0001
GradientThresholdMethod	12norm
GradientThreshold	inf
MaxEpochs	60
MiniBatchSize	256
Verbose	1
VerboseFrequency	50

References

1. Fan, X. et al. Ultrathin, rollable, paper-based triboelectric nanogenerator for acoustic energy harvesting and self-powered sound recording. ACS Nano 9, 4236–4243 (2015).

2. Yang, J. et al. Eardrum-Inspired active sensors for self-powered cardiovascular system characterization and throat-attached anti-interference voice recognition. Adv. Mater. 27, 1316–1326 (2015).

3. Liu, Y. et al. Epidermal mechano-acoustic sensing electronics for cardiovascular diagnostics and human-machine interfaces. Sci. Adv. 2, e1101185 (2016).

4. Ravenscroft, D. et al. Machine learning methods for automatic silent speech recognition using a wearable graphene strain gauge sensor. Sensors 22, 299 (2021).

5. Deng, C. et al. Ultrasensitive and highly stretchable multifunctional strain sensors with timbre-recognition ability based on vertical graphene. Adv. Funct. Mater. 29, 1–11 (2019).

6. Jin, Y. et al. Deep-learning-enabled MXene-based artificial throat: toward sound detection and speech recognition. Adv. Mater.Technol. 2000262, 2000262 (2020).

7. Qiu, L. et al. Ultrafast dynamic piezoresistive response of graphene-based cellular elastomers. Adv. Mater. 28, 194–200 (2016).

8. Lang, C., Fang, J., Shao, H., Ding, X. & Lin, T. High-sensitivity acoustic sensors from nanofibre webs. Nat. Commun. 7, 1–7 (2016).

9. Lee, J. H. et al. Highly sensitive stretchable transparent piezoelectric nanogenerators. Energy Environ. Sci. 6, 169–175 (2013).

10. Yang, T., Wang, W., Huang, Y., Jiang, X. & Zhao, X. Accurate monitoring of small strain for timbre recognition via ductile fragmentation of functionalized graphene multilayers. ACS Appl. Mater. Interfaces 12, 57352–57361 (2020).

11. Park, B. et al. Dramatically enhanced mechanosensitivity and signal-to-noise ratio of nanoscale crack-based sensors: effect of crack depth. Adv. Mater. 28, 8130–8137 (2016).

12. Kang, D. et al. Ultrasensitive mechanical crack-based sensor inspired by the spider sensory system. Nature 516, 222–226 (2014).

13. Tao, L. Q. et al. An intelligent artificial throat with sound-sensing ability based on laser induced graphene. Nat. Commun. 8, 14579 (2017).

14. Gong, S. et al. Hierarchically resistive skins as specific and multimetric on-throat wearable biosensors. Nat. Nanotechnol. 18, 889–897 (2023).

15. Qiao, Y. et al. Electromyogram-strain synergetic intelligent artificial throat. Chem. Eng. J. 449, 137741 (2022).

16. Yang, Q. et al. Mixed-modality speech recognition and interaction using a wearable artificial throat. Nat. Mach. Intell. 5, 169–180 (2023).

17. Liu, H. et al. An epidermal sEMG tattoo-like patch as a new human-machine interface for patients with loss of voice. Microsyst. Nanoeng. 6, 16 (2020).

18. Kang, Y. J. et al. Soft skin-interfaced mechano-acoustic sensors for real-time monitoring and patient feedback on respiratory and swallowing biomechanics. NPJ Digit. Med. 5, 147 (2022).

19. Jeong, H. et al. Differential cardiopulmonary monitoring system for artifact-canceled physiological tracking of athletes, workers, and COVID-19 patients. Sci. Adv. 7, eabg3092 (2021).
20. Lee, K. et al. Mechano-acoustic sensing of physiological processes and body motions via a soft wireless device placed at the suprasternal notch. Nat. Biomed. Eng. 4, 148–158 (2020).
21. Liu, Y. et al. Epidermal mechano-acoustic sensing electronics for cardiovascular diagnostics and human-machine interfaces. Sci. Adv. 2, e1601185 (2016).
22. Mehta, D. D., Zanartu, M., Feng, S. W., Cheyne, H. A. 2nd & Hillman, R. E. Mobile voice health monitoring using a wearable accelerometer sensor and a smartphone platform. IEEE Trans. Biomed. Eng. 59, 3090–3096 (2012).
