# Supplementary material for: Machine learning-assisted wearable sensing systems for speech recognition and interaction
Source: Nat Commun. 2025 Mar 10;16:2363. doi: 10.1038/s41467-025-57629-5 (PMC11894117; doi:10.1038/s41467-025-57629-5)
Supplement: Supplementary file 2 — Description of Additional Supplementary Files [file 41467_2025_57629_MOESM2_ESM.pdf]

Description of additional supplementary files

Supplementary Movie S1: Two participants demonstrated identity recognition applications using the fabricated device

Supplementary Movie S2: Lag of the Signal test of the fabricated device

Supplementary Movie S3: Participants used the fabricated device to control the Pac-Man games

Supplementary Movie S4: Participants used the fabricated device to control the robot dog to perform activities
